# Theory, the Final Frontier? A Corpus-Based Analysis of the Role of Theory in Psychological Articles

**DOI:** 10.3389/fpsyg.2017.00951

**Published:** 2017-06-08

**Authors:** Sieghard Beller, Andrea Bender

**Affiliations:** Department of Psychosocial Science, Faculty of Psychology, University of BergenBergen, Norway

**Keywords:** psychology, philosophy of science, theory, model, simulation, mechanism, law, hypothesis

## Abstract

Contemporary psychology regards itself as an empirical science, at least in most of its subfields. Theory building and development are often considered critical to the sciences, but the extent to which psychology can be cast in this way is under debate. According to those advocating a strong role of theory, studies should be designed to test hypotheses derived from theories (theory-driven) and ideally should yield findings that stimulate hypothesis formation and theory building (theory-generating). The alternative position values empirical findings over theories as the lasting legacy of science. To investigate which role theory actually plays in current research practice, we analyse references to theory in the complete set of 2,046 articles accepted for publication in *Frontiers of Psychology* in 2015. This sample of articles, while not representative in the strictest sense, covers a broad range of sub-disciplines, both basic and applied, and a broad range of article types, including research articles, reviews, hypothesis & theory, and commentaries. For the titles, keyword lists, and abstracts in this sample, we conducted a text search for terms related to empiricism and theory, assessed the frequency and scope of usage for six theory-related terms, and analyzed their distribution over different article types and subsections of the journal. The results indicate substantially lower frequencies of theoretical than empirical terms, with references to a specific (named) theory in less than 10% of the sample and references to any of even the most frequently mentioned theories in less than 0.5% of the sample. In conclusion, we discuss possible limitations of our study and the prospect of theoretical advancement.

## Introduction

Psychology shares with philosophy an interest in bold questions: Who are we? How do our minds work? Why do we behave the way we do? Where does consciousness come from and where does it reside? Or: Do we have a free will? Questions like these figure prominently in human reasoning and intellectual endeavors around the world (e.g., White and Kirkpatrick, 1987; Wierzbicka, 1989; Lillard, 1998; Scharfstein, 1998) and have fuelled scholarly discourse within European philosophy for over two millennia (Flanagan, 1991; Brysbaert and Rastle, 2013). Since the late 19th century, psychology has generated innumerable insights into the human mind and behavior. For instance, it has demonstrated that we find a boring task more interesting if we are paid less for taking part (Festinger and Carlsmith, 1959), that our mind changes when we learn a second language (Bialystok et al., 2012), or that social pressure can affect the way we behave in dramatic ways (Zimbardo, 2007).

Ever since psychology's evolution into a discipline in its own right, it has preferred to think of itself as a science rather than a field within the humanities, involving data collection based on unbiased observations or systematic experimentation, a goal to discover patterns or regularities in these data, and a concern with theoretical accounts that help organize, explain, and predict these patterns.

As a scientific endeavor, psychology has been facing major challenges, one of which relates to its subject: mind, brain, and social behavior of humans and other species. Psychology's quest for unbiased investigation is hampered by the subject's less tangible, more malleable, and highly responsive nature compared to many of the phenomena investigated in the natural sciences. Matters are complicated further by the fact that psychologists are humans themselves: They rely on mind, brain, and social behavior to investigate the very same, and are thus themselves participating in the phenomena they seek to explain. This confounding of researcher and researched not only renders the historical and culture-specific background of researcher and research a critical condition (e.g., Medin et al., 2010), but also leads to the paradox of a system trying to comprehend itself. To paraphrase a truism: The mind can only be understood if it is simple enough to be understood; but we can only hope to understand it if the mind is also complex enough to be able to understand.

Another major challenge faced by psychology is its capacity for, or concern with, theory building and development, which is often considered critical to the sciences. A theory is a systematic explanatory scheme on a greater scale than those patterns and regularities it attempts to account for, including an explanation for relations between them and for why they are obtained. The state of theory building in psychology has been an issue in the field from time to time (e.g., Watkins, 1984; Fiedler, 1991; Gigerenzer, 1991, 1998, 2010; Wallach and Wallach, 1994, 1998). It comes in three distinct, yet related forms, focusing on the degree of general interest in theory, the scope of existing theories, and the decision between competing theories for the same phenomena, respectively.

A concern focusing on a general disinterest in theory was put forward, for instance, by Gerd Gigerenzer stating that “psychology has no theory. It has many local ones but no overarching theory, not even a provisional one. Yet there is something even more surprising: a lack of awareness of the value of integration” (2010, p. 734). In the inaugural article for the specialty section on Theoretical and Philosophical Psychology, Dan Lloyd depicted the discipline as “the rollercoaster of the sciences. In its brief history, psychology has swung through many -isms, alternately embracing, and rejecting the widest variety of assumptions and first principles” (2010, p. 1). One of the grand challenges, he continued, will therefore be to stake out hypotheses and theories on autonomous grounds. And two years later, Shimon Edelman emphasized that only overarching psychological theories will help scientists make sense of their findings, thereby preventing entire fields from “decades-long wild goose chase” (2012, p. 2). Likewise, Stroebe and Strack (2014) argued that empirical outcomes are meaningful only in terms of the underlying theory (and see Dijksterhuis, 2014; Hommel and Colzato, 2017, for similar arguments).

There are indeed many scientists, who simply value data over theory, some even quite candidly. In their guidelines for “interesting research,” for instance, Gray and Wegner (2013) attribute the longevity of the work of Milgram, Asch, and Zimbardo not to their theories, but to the psychological weight of obedience, conformity, and cruelty. Likewise, Simons claims that “[a]ccumulated evidence for reliable effects is the lasting legacy of science—theories come and go” (2014, p. 79). And Greenwald (2012)—while not arguing against theory as a reference for research—emphasizes the crucial role played by methods both in scientific discoveries and in public esteem, as attested to, for instance, by the overwhelming majority of Nobel Prizes in physics, biology, and medicine being awarded to methodological inventions rather than to theoretical contributions.

But even when psychologists attempt to derive their hypotheses from theoretical considerations and to build theories from the data they collect, the scope of these theories has given rise to concerns. What may appear at first glance to be theories often turn out on closer inspection to be “surrogates for theories” only, such as circular restatements, one-word explanations, lists of vague dichotomies, or instances of data fitting (Gigerenzer, 1998, 2010). According to Gigerenzer, this is partly due to a disregard of first principles in building theories (e.g., accuracy, consistency, broad scope, simplicity, and fruitfulness), including an abundant use of free parameters, but is very frequently owing to a lack of training on how to build theories to begin with (Tesser, 2000). In the most severe cases, these practices might not only hamper the building of powerful and precise theories, but might reverse the development from already existing theories to surrogates (Gigerenzer, 2010). This is often contrasted with the situation in biology, where the theory of evolution is seen as such a fundamental keystone of theorizing that “nothing in biology makes sense, except in the light of evolution” (Dobzhansky, 2013)—and even one that has appealed as a theoretical framework to psychology itself (Stam, 2015; and see Lloyd, 2010; Edelman, 2012).

Finally, even when researchers are concerned with theory building on a larger scale, theoretical advancement is hampered by the persistence of competing theories on the same phenomenon, in spite of decades of research into it (Greenwald, 2012), as can be seen in the case of theories on mental models (Johnson-Laird et al., 1992, 1994) vs. mental rules (O'Brien et al., 1994; Rips, 1994) for human reasoning (for more examples, see Greenwald, 2012). The publication bias, among other dubious practices, makes it almost impossible to falsify theories in psychology (Ferguson and Heene, 2012), and if a theory is eventually abandoned it is often not because it has been disproven but because it no longer matches a prevailing paradigm or because researchers have simply lost interest in it. In this vein, both mental model theory and mental rule theory are now losing ground in what is called “the new paradigm” of reasoning (Over, 2009), which adopts a Bayesian probabilistic approach instead of an approach based on logic or propositions.

Theoretical advancement presupposes abandonment of less convincing accounts and the eventual integration of a plethora of small-scale theories into larger theories. Currently, there are several conditions which foster both the persistence of competing theories and the branching of small-scale theories into even smaller twigs. One is known as the “toothbrush problem”: Theories are often treated as someone else's toothbrush—fine for that individual to use, but unbefitting for the rest (Watkins, 1984; and quoted in Gigerenzer, 2010). This reluctance to use anyone else's theories is nowadays amplified by the request of tenure committees, funding organizations, and editorial boards to deliver *original* ideas, models, and theories that do *not* build on anyone else's work (Mischel, 2009).

Regardless of the position one takes in the debate on whether theory is the key or a barrier to scientific advance in psychology, there is a lack of empirical data on the degree to which authors nowadays actually do rely on theory or attempt to revise it. Against this background, we aim to take a snapshot of the role played by theory in contemporary psychology, and we do so using the following proxies: (i) we take what psychologists publish in journal articles (arguably their most valued currency) as a reflection of their concern with a specific topic, in this case theoretical frameworks; (ii) we take a sample of more than 2,000 such articles—published in 2015 in a journal of psychology that is not restricted to specific topics and that claims to be “The #1 largest and the #2 most cited Psychology journal,” namely *Frontiers in Psychology*—as a largely representative sample of work in scientific psychology; (iii) we take the mention of theory-related terms in the titles, keywords, and abstracts of these articles as indicative of the relevance that they grant to theory (as compared to the mention of empirical terms); and (iv) we take a selection of six terms as comprehensive evidence of this concern. Clearly, each of these proxies has its limitations, and we discuss these limitations critically in the final part of this paper, which is also devoted to the prospects of theoretical advancement.

## The sample of articles

The analysis is based on the complete set of 2,046 articles accepted for publication in *Frontiers in Psychology* in 2015 (with DOIs ranging from 10.3389/fpsyg.2015.00001 to 10.3389/fpsyg.2015.02066, of which 20 DOIs do not refer to an article^1^); a total of 1,914 of these articles were also published in 2015, with the remainder being published in 2016. Since its launch in 2010, *Frontiers in Psychology* has published 8,674 articles (by May 2nd 2017); our sample thus represents about one quarter of the total number of articles published so far.

### Accessed information

The *Frontiers* editorial office provided us with an Excel sheet comprising the following information on each article: *specialty section* and *research topic* (if applicable) in which the article appeared, *article type, title, authors' names, acceptance date, publication date, DOI*, and a link to the respective *Frontiers webpage*. We then accessed each article through its DOI and copied the *keywords* (for all articles) as well as the *abstract* (if applicable) into the Excel sheet.

Whether or not a *Frontiers* article includes an abstract depends on the article type (see Table 1). 85.9% (1,757) of the articles in our sample had an abstract. Abstracts that consisted of a single paragraph (1,698) were copied in their original form. A small number of abstracts (59) consisted of several paragraphs, each introduced by a “subheading,” typically a selection of *aim, background, introduction, objective, purpose, approach, design, methodology, methods, sample, findings, results, conclusions, discussion*, and *implications*. In these cases, we deleted the subheadings (as they simply structure the abstract, but do not contribute to its content) and concatenated the paragraphs to form a single paragraph before copying the abstract.

**Table 1:** Abstract (yes/no) and number of articles (*n* and %) of each article type.

**Rank and article type**	**Abstract**	**Number of articles**	
1. Original Research	Yes	1,351	66.0%
2. Opinion	No^a^	133	6.5%
3. Review	Yes	130	6.4%
4. Hypothesis & Theory	Yes	107	5.2%
5. General Commentary	No	89	4.3%
6. Perspective	Yes	69	3.4%
7. Mini Review	Yes	49	2.4%
8. Editorial	No	45	2.2%
9. Methods	Yes	36	1.8%
10. Book Review	No	7	0.3%
Erratum	No	7	0.3%
11. Clinical Case Study	Yes	6	0.3%
12. Frontiers Commentary	No	3	0.1%
Specialty Grand Challenge	No	3	0.1%
Technology Report	Yes	3	0.1%
13. Case Report	Yes	2	0.1%
Correction	No	2	0.1%
Data Report	Yes^b^	2	0.1%
14. Evaluation	Yes	1	0.05%
Focused Review	Yes	1	0.05%
		2,046	100.0%

a *One Opinion included a “précis,” which was counted as an abstract*.

b *One Data Report did not include an abstract*.

### Description of the sample

Six variables are used for further analyses: *specialty section, research topic, article type, title, keywords*, and *abstract*. This section provides a descriptive overview of the variables.

*Frontiers* articles appear in different subsections or *specialty sections* of a journal. By May 2nd 2017, *Frontiers in Psychology* comprised 27 specialties. Our sample of 2,046 articles is spread across 24 of these specialties. The top three specialties, with the largest number of accepted articles in 2015, are *Cognition* (16.9%), *Cognitive Science* (11.1%), and *Language Sciences* (10.4%). The one specialty with “theory” in its name—*Theoretical and Philosophical Psychology*—ranks 18th (1.1%). Submissions to this section are expected to foster interdisciplinary exchange between psychology and philosophy, including the discussion of psychology as a science “from the point of view of philosophy of science,” thereby promoting meta-theoretical perspectives on the field. The complete distribution is shown in Table 2.

**Table 2:** Number of articles (n and %) for each specialty section.

**Rank and specialty section**	**Number of articles**	
1. Cognition	345	16.9%
2. Cognitive Science	228	11.1%
3. Language Sciences	213	10.4%
4. Developmental Psychology	160	7.8%
5. Psychology for Clinical Settings	148	7.2%
6. Emotion Science	108	5.3%
7. Educational Psychology	104	5.1%
8. Personality and Social Psychology	100	4.9%
9. Perception Science	96	4.7%
10. Quantitative Psychology and Measurement	88	4.3%
11. Psychopathology	77	3.8%
12. Consciousness Research	66	3.2%
13. Auditory Cognitive Neuroscience	56	2.7%
14. Movement Science and Sport Psychology	44	2.2%
15. Comparative Psychology	31	1.5%
16. Evolutionary Psychology and Neuroscience	28	1.4%
17. Organizational Psychology	27	1.3%
18. Theoretical and Philosophical Psychology	23	1.1%
19. Eating Behavior	22	1.1%
Performance Science	22	1.1%
20. Decision Neuroscience	16	0.8%
21. Psychoanalysis and Neuropsychoanalysis	15	0.7%
22. Cultural Psychology	14	0.7%
23. Human-Media Interaction	11	0.5%
(No specialty)	4	0.2%
	2,046	100.0%

Many *Frontiers* articles are part of a special issue or *research topic*—a collection of articles on a specific topic—which is also made available as an “ebook” upon completion. By May 2nd 2017, *Frontiers in Psychology* comprised 509 research topics. Among the articles of our sample, 57.5% (1,176) are part of such a research topic, distributed over 194 different topics. The number of articles per research topic ranges between 1 and 26 (*m* = 5.86; *SD* = 5.21).

When submitting to *Frontiers*, authors have to decide on an *article type*, which determines, among other factors, the length of the article, the inclusion of an abstract, and the publication fee. Our sample of 2,046 articles is spread across 20 article types, with *Original Research* articles being strongly prevalent (66.0%). The one article type with “theory” in its name—*Hypothesis* & *Theory*—ranks 4th (5.2%). Submissions in this category are expected to “present a novel argument, interpretation or model intended to introduce a new testable hypothesis or theory or support already existing theories.” The complete distribution is shown in Table 1 (on the previous page).

With regard to the *title* of an article, the American Psychological Association (APA) recommends a length of “no more than 12 words” (American Psychological Association, 2010, p. 23). Only 50.4% (1,031) of the articles in our sample meet this criterion. The titles are a little longer on average (*m* = 12.8 words) and show considerable variation (*range:* 2–30; *SD* = 4.4).

**Figure 1:**
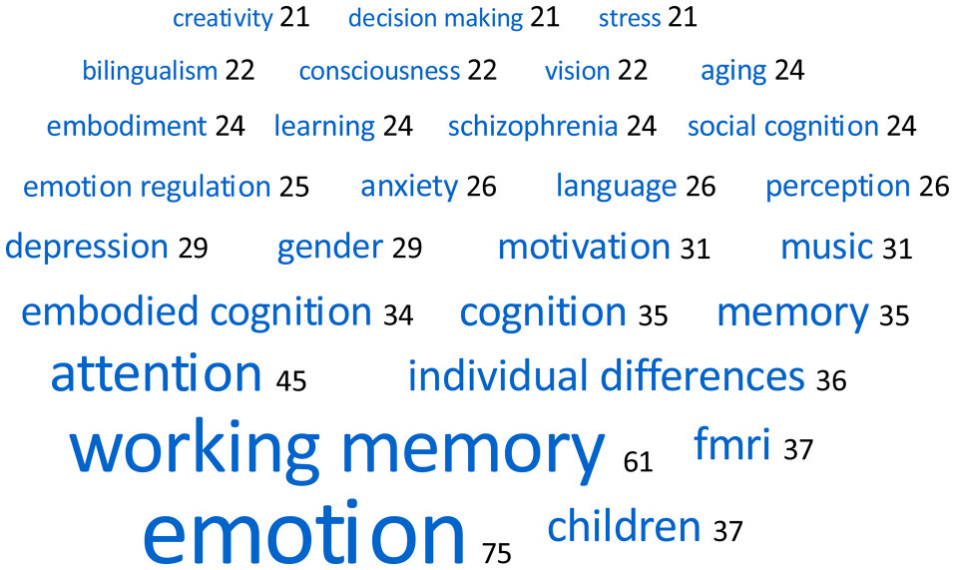
Word cloud of the 28 keywords that are shared by more than 20 (1%) of the 2,046 articles.

All *Frontiers* articles include a list of *keywords*. Defining “keywords” as entries separated by commas, our sample of articles comprises a total of 11,842 keywords (*m* = 5.8 keywords per article; *range:* 2–12; *SD* = 1.17). If we ignore uppercase and lowercase writing and remove duplicates of otherwise identical keywords, this number can be condensed to 7,008 *different* keywords. Among these, 78.2% (5,479) keywords are used in one article only. Authors appear to choose their keywords highly specifically, if not idiosyncratically. An overview of the keywords that are shared by more than 1% of the articles in our sample (i.e., more than 20 times) is provided as a word cloud in Figure 1. All of these refer to psychological phenomena, except for two: *children* refers to a specific age group, and *fmri* to a method. The top three keywords are *emotion* (shared by 3.7% of the articles), *working memory* (3.0%), and *attention* (2.2%).

Finally, with regard to the length of an *abstract*, the APA indicates a range from 150 to 250 words as typical for psychology journals (American Psychological Association, 2010, p. 27). The average abstract in our sample of articles falls into this range (*m* = 209.5 words), but with considerable variation (*range:* 58–484; *SD* = 57.1). Among the total of 1,757 abstracts, 15.1% (266) have less than 150 words, and 25.8% (453) have more than 250 words.

## Three levels of analysis

Even from the description of the sample in the previous section, we might already derive two slightly different assessments of the status of theory in psychology: On the one hand, the article type *Hypothesis* & *Theory* ranked 4th out of 20 categories, indicating that on this level, authors consider dealing with theory to be an important concern. On the other hand, the specialty *Theoretical and Philosophical Psychology* ranked only 18th out of 24 categories and was thus one of several rather small specialties in 2015 (despite it being among the original specialties of *Frontiers in Psychology*, Lloyd, 2010). However, article types and specialties are rather superficial criteria, which provide only a coarse-grained assessment of the status of theorizing in psychology. A more substantive answer is to be found in the content of articles.

### Basic text search

In a first step, we collected a broad range of search strings that are potentially related to the two categories *empiricism* or *theory*, for example ^*^result^*^ and ^*^effect^*^ (empiricism) vs. ^*^model^*^ and ^*^hypothe^*^ (theory). We used asterisks to indicate that the respective string can be preceded or followed by any series of characters, in order to be able to cover a broad range of contexts and possible terms (e.g., hypothesis, hypotheses, hypothesize, hypothesize, and hypothetical). In most cases, single strings were used for the search (e.g., ^*^result^*^); in two cases, we combined two strings using a logical “or” to enable to cover different spellings. As motivated in the introduction, we restricted our search to the titles, keywords, and abstracts of the articles in our sample, which should be indicative of their content. Finally, we counted separately in how many titles, keyword lists, and abstracts each search string matched (using Excel's text filter function “contains”), and calculated in how many articles each string matched at least once in any of the categories *title, keyword list*, or *abstract*. An overview of the search strings and the respective frequencies is shown in Table 3.

**Table 3:** Percentage of the titles, keyword lists, and abstracts, in which a search string matched, as well as percentage of articles with at least one match in any of the three categories title, keyword list, or abstract.

**Search string**	**Titles (*N* = 2,046)**	**Keyword lists (*N* = 2,046)**	**Abstracts (*N* = 1,757)**	**At least in one (*N* = 2,046)**
^*^study/-die^*^^a^	6.9 ^(2)^	1.4 ^(10)^	65.5 ^(1)^	58.1 ^(1)^
^*^result^*^	0.5 ^(20)^	0.1 ^(22)^	53.9 ^(2)^	46.5 ^(2)^
^*^effect^*^	10.4 ^(1)^	5.4 ^(3)^	40.8 ^(3)^	39.1 ^(3)^
^*^participa^*^	0.4 ^(21)^	0.2 ^(19)^	37.6 ^(4)^	32.4 ^(4)^
^*^finding^*^	0.4 ^(22)^	0.05 ^(23)^	31.2 ^(5)^	27.0 ^(5)^
^*^task^*^	4.4 ^(3)^	4.2 ^(5)^	28.7 ^(6)^	25.7 ^(6)^
^*^test^*^	2.2 ^(8)^	2.3 ^(7)^	28.2 ^(7)^	25.5 ^(7)^
^*^analys/-lyz^*^^a^	3.4 ^(5)^	4.9 ^(4)^	25.3 ^(8)^	24.0 ^(8)^
^*^experiment^*^	0.6 ^(19)^	0.9 ^(14)^	24.0 ^(9)^	21.1 ^(9)^
^*^theor^*^	3.2 ^(6)^	6.0 ^(2)^	20.5 ^(10)^	21.1 ^(10)^
^*^model^*^	3.4 ^(5)^	6.3 ^(1)^	20.3 ^(11)^	19.0 ^(11)^
^*^evidence^*^	3.6 ^(4)^	0.4 ^(18)^	18.3 ^(13)^	18.3 ^(12)^
^*^predict^*^	3.2 ^(6)^	1.9 ^(8)^	18.8 ^(12)^	17.4 ^(13)^
^*^data^*^	1.2 ^(15)^	0.6 ^(16)^	17.5 ^(14)^	15.5 ^(14)^
^*^hypothe^*^	0.7 ^(17)^	1.2 ^(11)^	15.1 ^(15)^	13.8 ^(15)^
^*^approach^*^	2.5 ^(7)^	1.1 ^(13)^	12.6 ^(18)^	12.3 ^(16)^
^*^expla^*^	0.9 ^(16)^	0.4 ^(18)^	13.1 ^(16)^	11.7 ^(17)^
^*^method^*^	1.3 ^(12)^	2.3 ^(7)^	11.3 ^(20)^	11.6 ^(18)^
^*^caus^*^	1.2 ^(14)^	1.1 ^(12)^	12.7 ^(17)^	11.5 ^(19)^
^*^mechanis^*^	1.3 ^(13)^	0.6 ^(16)^	11.8 ^(19)^	10.7 ^(20)^
^*^concept^*^	1.5 ^(11)^	1.9 ^(9)^	9.6 ^(22)^	9.4 ^(21)^
^*^account^*^	0.7 ^(18)^	0.1 ^(22)^	9.8 ^(21)^	8.8 ^(22)^
^*^bias^*^	1.6 ^(9)^	2.9 ^(6)^	7.1 ^(23)^	7.3 ^(23)^
^*^perspective^*^	1.6 ^(10)^	0.7 ^(15)^	6.8 ^(24)^	6.9 ^(24)^
^*^phenomen^*^	0.5 ^(20)^	0.4 ^(18)^	5.5 ^(26)^	5.0 ^(25)^
^*^assum^*^	0.1 ^(26)^	0.0 ^(24)^	5.7 ^(25)^	4.9 ^(26)^
^*^framework^*^	0.3 ^(23)^	0.1 ^(21)^	4.9 ^(27)^	4.3 ^(27)^
^*^princip^*^	0.2 ^(25)^	0.4 ^(17)^	2.9 ^(28)^	2.9 ^(28)^
^*^simulat^*^	0.2 ^(24)^	0.7 ^(15)^	2.8 ^(29)^	2.8 ^(29)^
^*^law^*^	0.2 ^(24)^	0.2 ^(20)^	1.1 ^(30)^	1.1 ^(30)^

An asterisk (^*^) can represent any series of characters. Uppercase numbers indicate the ranks of the search strings in that column.

a Two strings combined with a logical or: ^*^study^*^ or ^*^studie^*^; ^*^analys^*^ or ^*^analyz^*^.

The distributions exhibit a large variability in the frequencies of occurrence, ranging for the titles from 0.1% (^*^assum^*^) to 10.4% (^*^effect^*^), for the keyword lists from zero (^*^assum^*^) to 6.3% (^*^model^*^), for the abstracts from 1.1% (^*^law^*^) to 65.5% (^*^study/-die^*^), and across the three categories from 1.1% (^*^law^*^) to 58.1% (^*^study/-die^*^). The top three search strings matched empirical terms for the abstracts and across the three categories (^*^study/-die^*^, ^*^result^*^, and ^*^effect^*^) as well as for the titles (^*^effect^*^, ^*^study/-die^*^, and ^*^task^*^). Interestingly, for the keywords, the top three search strings included only one string matching empirical terms (^*^effect^*^), while the other two matched theoretical terms (^*^model^*^ and ^*^theor^*^).

This kind of basic text search provides a first content-based approximation of the relevance of theory in psychological articles. With search strings like ^*^result^*^, ^*^effect^*^, ^*^finding^*^, ^*^task^*^, and ^*^experiment^*^ all being among the top-ranked (matching in substantial proportions ranging between 20 and 60% of the articles at least once in any of the titles, keyword lists, or abstract), the results seem to confirm that current psychological research in the represented subfields is strongly empirical in nature. Theory also seems to play a role—the two strings ^*^theor^*^ and ^*^model^*^ ranked #10 and #11, each matching in about 20% of the articles—but this role is much weaker.

However, the search strings used for this basic text search might include meanings that are not relevant for our purpose. The search string ^*^law^*^, for example, matches not only with scientific laws, but also with terms like “lawyer” or “flaw.” Similarly, the string ^*^theor^*^ matches not only with scientific theories, but also, among others, with terms like “theory of mind,” which refers to the ability for mental state reasoning and a cluster of related phenomena that in turn are explained by competing theories such as the theory-theory or simulation-theory (Gopnik and Wellman, 1992; Stich and Nichols, 1992; vs. Gordon, 1986; and see Apperly, 2008). The numbers from this analysis thus provide an upper bound of occurrences rather than a precise assessment; we therefore need to assess the exact referents of each match and analyse their usage and meaning in more detail; this is undertaken for a selection of terms in the next section.

### Usage and meaning of selected terms

The further analysis was restricted to six terms, *hypothesis, law, mechanism, model, simulation*, and *theory* (assessed through the respective search strings ^*^hypothe^*^, ^*^law^*^, ^*^mechanis^*^, ^*^model^*^, ^*^simulat^*^, and ^*^theor^*^). Theory was included because it is the concept of prime interest to this investigation^2^. *Hypotheses* on as yet unproven relations between variables are an essential part of theory evaluation and in some instances may even gain theory-like status such as the “Sapir-Whorf Hypothesis.” *Laws, mechanisms, models*, and *simulations*, finally, serve as intermediate steps between hypotheses and theories, building on each other in a hierarchical manner. Compared to hypotheses, laws describe verified regularities in the world. Mechanisms add a causal underpinning and have become a much more relevant component in psychological theories than laws (e.g., Wright and Bechtel, 2007). Models serve as a manipulable representation of a phenomenon that cannot be observed directly and are therefore a central component of modern science typically used for simulation purposes. While they form abstractions, like theories do, the latter should add a broader explanatory framework (Westermann, 2000). Focusing on these terms is also justified by the frequency of occurrence in the sample. In the basic text search, ^*^theor^*^ was the most frequently used string referring to a potentially theoretical meaning, followed by ^*^model^*^. The strings ^*^hypothe^*^ and ^*^mechanis^*^ were at a medium level among the searched strings, whereas ^*^simulat^*^ and ^*^law^*^ were in the lowest range. Still, considering these six search strings jointly should allow us to cover a broad range of theory-related meanings (for possible implications of this selection, see also the discussion in Section Limitations by Proxy).

In the following, we refine our analysis for these six search strings. We searched each string across the titles, keyword lists, and abstracts, inspected each match, and classified the article according to whether at least one match referred to a theoretical meaning. In a few cases, in which neither the title, nor the keywords, nor the abstract was sufficiently explicit to decide upon the usage and meaning of a matching term, we checked the full article for clarification. One of the analyzed search strings (^*^hypothe^*^) was categorized by two people independently to assess interrater reliability (3 categories, 306 classifications for 282 articles; see Section Hypotheses). With an agreement of 95.8% and a Cohen's kappa of 0.891, the results were excellent. The remainder of the categorization was therefore completed by one rater.

#### Hypotheses

A *hypothesis* claims a relation between variables that needs empirical evaluation and hence is only a first step on the way to a theory. Hypotheses derived from theories may play an essential role in theory evaluation. In some instances, a specific hypothesis may be so important for driving a whole research field that it gains a theory-like status while retaining its original label (especially when it is still controversial). A prominent example is the “Sapir-Whorf Hypothesis,” also known as “Linguistic Relativity Hypothesis” (Whorf, 1956; and see Wolff and Holmes, 2011; Cibelli et al., 2016).

The search string ^*^hypothe^*^, used for the basic text search, matched in 15 titles, 25 keyword lists, and 266 abstracts of a total of 282 of the 2,046 articles (13.8%). We classified each instance according to its usage and meaning as referring either to at least one named scientific hypothesis, to scientific hypothesis testing in general (without naming a specific hypothesis), or to another meaning that is irrelevant for our present purpose. In 13 articles, ^*^hypothe^*^ matched solely terms with such an irrelevant meaning (e.g., hypotheses that participants of a study generate in a task, the brain's capacity to generate hypotheses, or “hypothesis and theory article”). In the 269 other articles (13.1% of the sample), the search string ^*^hypothe^*^ matched terms that were used to refer to a hypothesis in a scientific sense at least once.

Of these 269 articles, 216 articles (80.3%) made only a reference to hypothesis testing in general, either to a claimed relation between variables (e.g., “It has been hypothesized that mental addition leads to rightward and upward shifts of spatial attention” or “supporting the hypothesis that Spanish speakers are affected by interferences from their L1”) or to statistical procedures for hypothesis testing (e.g., “null hypothesis test”). Only 53 articles (19.7%) named at least one hypothesis, such as “the buffering hypothesis” or “the Interaction Engine Hypothesis.” Most of these articles (44) named only one hypothesis. Only 9 articles named two or three hypotheses, indicating that comparisons of competing hypotheses were not that common in our sample of articles.

Altogether, 56 *different* hypotheses were named; 51 hypotheses in one article only, four hypotheses in two articles (the body-specificity hypothesis, the continuity hypothesis, the sexualized-body-inversion hypothesis, and the somatic markers hypothesis) and one hypothesis in four articles (the uncanny valley hypothesis). Whether or not such names refer to already established theories in the respective research field (despite the label “hypothesis”) is difficult to decide. Some names suggest that the hypothesis refers to a (claimed) cognitive mechanism (e.g., the buffering hypothesis, the integration hypothesis, or the structuring hypothesis), while other names seem to refer to more general aspects (e.g., the common cause hypothesis, the less-is-more hypothesis). Still other names are used by some authors in an idiosyncratic manner for the sole purpose of facilitating the descriptive distinction between alternative hypotheses.

#### Laws

According to some classic definitions, *scientific laws* belong to the basic cornerstones of theories. They describe systematic relations between variables of a system that hold under specific circumstances and apply to specific situations. A classic example would be the 2nd law of thermodynamics on entropy in physics. Laws can be used to predict the behavior of the system, and are often claimed to be universal. In contrast to hypotheses, laws are well-established on empirical grounds. Although, a causal relation is usually assumed to generate the regularities, the causal mechanism need not be known. Although, in psychological theorizing Wright and Bechtel (2007) notice a shift in the emphasis from laws to mechanisms, the term is included here due to its historical relevance (for examples, see below).

The search string ^*^law^*^, used for the basic text search, matched in five titles, four keyword lists, and 19 abstracts of a total of 23 of the 2,046 articles (1.1%). We classified each instance as referring either to a named scientific law, to scientific laws in general (without naming a specific law) or to another (irrelevant) meaning. In eleven articles, ^*^law^*^ matched solely terms with an irrelevant meaning (e.g., law in a juridical sense, “Malawi,” “flaw,” or “lawn mower”). In the 12 other articles (0.6% of the sample), the search string ^*^law^*^ matched the term *law* in a scientific meaning.

Of these 12 articles, two articles (16.7%) made only a general reference to laws (perception being regarded as “constituted by the mastery of lawful sensorimotor regularities” and psychological measurement being regarded as the “foundation of quantitative theory, definition and law”), while 10 articles (83.3%) named a specific law. Four laws were related to perception (*Fitt's law, Weber's law, law of good continuation*, and *perceptual laws of tonal gravity*); two were related to learning (*power law of learning*, and *generalized matching law*), and one each to arousal (*Yerkes-Dodson law*), motivation (*law of least effort*), decision making (*law of small numbers*), and physics (*laws of thermodynamics*).

#### Mechanisms

*Mechanism* is a concept that goes beyond the acknowledgement of regularities by adding explanation. Being related to physical mechanics, and especially machines, it typically implies a deterministic (causal) underpinning on a material basis, which can be used to account for and predict the functioning or malfunctioning of a system from its parts. It is often used in a reductionist manner insofar as a phenomenon on one level (e.g., a clinical syndrome) is reduced to causally connected parts on another, “lower” level (e.g., a neural structure), but increasingly often also in a broader sense of any causal relation that can explain the phenomenon. According to Wright and Bechtel (2007), explanations of psychological phenomena in terms of mechanisms have replaced those concerned with laws, and mechanistic accounts generate interfield theories that bridge levels of explanations.

The search string ^*^mechanis^*^ matched in 26 titles, 13 keyword lists, and 208 abstracts of a total of 219 of the 2,046 articles (10.7%). We classified each instance as referring either to a mechanism for explaining a psychological phenomenon or to another (irrelevant) meaning. Only in five articles were the matches not directly related to psychological explanation, but rather referred to a measurement tool (“transcripts were coded with the Defense Mechanisms Rating Scale,” fMRI technology as an “effective mental sub-health warning mechanism”) or to cognitive processes in a general way (“The human body, as the delivery mechanism of communication,” a program that includes a “core training of target cognitive mechanisms,” “the language faculty has been shrinking—perhaps to include only the mechanism of recursion”). In the 214 other articles (10.5% of the sample), the term *mechanism* was related to the explanation of a psychological phenomenon.

However, this does not mean that a concrete mechanism was specified in each of these cases. On the contrary: Many statements simply pointed out that a mechanism for the phenomenon at hand was still lacking (e.g., “The exact mechanisms by which prosodic cues enhance learning are fully unknown”). The matches also differed with regard to the type of mechanism they described: While some referred to an underlying neural mechanism, others referred to a set of explanatory variables—and in the most extreme cases, only to one such variable (e.g., “it investigates trust as a mediating mechanism”)—or to a group of psychological processes (e.g., “domain-general attention mechanism”).

#### Models and simulations

*Models* are abstractions of a section of reality. They simplify a subject matter, especially one that cannot be observed directly, and stand in a representational relation to it insofar as specific parts of the model map to specific parts of the subject matter. Models can be realized in substance or symbolically, and they can represent a concrete mechanism or simply connect a set of variables; in either case they are intended as a tool to *simulate* the behavior of a system under varying conditions. We therefore looked at two search strings here, ^*^model^*^ and ^*^simulat^*^.

The search string ^*^model^*^ matched in 69 titles, 128 keyword lists, and 356 abstracts of a total of 389 of the 2,046 articles (19.0%). We classified each instance as referring either to a model that represents a psychological phenomenon (or empirical data on such a phenomenon) or to another (irrelevant) meaning. In 30 articles, the terms *model* or *modeling* were used in an irrelevant meaning, referring either to a person's internal mental model of something (e.g., “shared mental models” or “crucial for the brain in building a useful model of the distal world”), to an experimental situation serving as model for a real-world situation (e.g., “[the] fear conditioning paradigm may serve as an ecologically valid laboratory model for unexpected panic attacks”), to a person serving as model for the participant (e.g., “using pictures of female models” or “learning from either a live or televised model”), or to a specific type of object (e.g., “we characterized a number of LCD monitors to determine if newer models are suitable”). In the 359 other articles (17.5% of the sample), the model represented a psychological phenomenon or data on such a phenomenon.

Modeling techniques included, among others, mathematical or statistical models^3^ (such as structural equation models, diffusion models, and Bayesian models), other variants of computational models (such as connectionist and ACT-R models), animal models (e.g., “translating an animal model for social fear conditioning [SFC] to a human sample”), neuro-cognitive models (e.g., “Koelsch's neurocognitive model of music perception”), and verbally described models (e.g., “The interactive influence model of emotion and cognition”), but also more general references to models or the lack of models (e.g., “models of reading should incorporate this aspect” and “lack of suitable empirical theory or model to guide design strategies”).

Like ^*^law^*^, the search string ^*^simulat^*^ was one that produced only a few matches: in five titles, 15 keyword lists, and 50 abstracts of a total of 58 of the 2,046 articles (2.8%). We classified each instance as referring to one of three types of scientific simulation techniques or to another (irrelevant) meaning. In 14 articles, simulation was solely used in an irrelevant meaning, referring to mental simulation as an internal process involved in embodied cognition or Theory of Mind phenomena (e.g., “sensorimotor experiences in the form of ‘re-enactments’ or ‘simulations’ …” or “the ‘simulationist’ account assumes that affect sharing is involved in recognizing emotion”). Here, the simulation is part of the psychological phenomenon to be explained, and is therefore not relevant for our purpose. In the 44 other articles (2.2% of the sample), simulations were used as scientific method.

Among these 44 articles, three different types of usage could be distinguished: (a) In 20 cases, the simulation concerned the establishment of experimental conditions (e.g., “simulated natural listening environments” or “simulated tactile feedback”). Here, the simulation is used by the scientists to model real-world situations; it does not refer to a psychological phenomenon, but at best to its framing conditions. (b) In nine cases, the simulation concerned an evaluation of statistical procedures (as in, e.g., a simulation that “demonstrate[s] the dominance of hierarchical models over rANOVA”). Here, simulations are used to advance methods for data analysis. (c) In the final 15 cases, simulation referred to psychological phenomena, for instance, of learning, gender categorization, language acquisition, and decision making.

#### Theories

A *scientific theory* refers to a section of reality in a similar way to a model: It abstracts from reality, simplifies it, and corresponds with particular aspects of reality. A theory is much broader in scope, however, encompassing a whole family of regularities or laws, and goes beyond simple representation by adding explanation. As abstract, ideational structures, theories have to undergo empirical testing, for which specific hypotheses are derived. The question of empirical adequacy or validity arises in a similar way for models and to some extent also for the description of mechanisms and laws, but it is discussed more prominently in relation to hypotheses and theories (Popper, 1959; Stroebe and Strack, 2014).

Of the six search strings analyzed in more detail in this section, the string ^*^theor^*^ matched most often: in 65 titles, 123 keyword lists, and 361 abstracts of a total of 431 of the 2,046 articles (21.1%). We classified each instance as referring either to at least one named theory, to theory in general (without naming a specific theory), or to another (irrelevant) meaning. In 39 articles, the term *theory* was only used in an irrelevant meaning, referring in almost all cases to a person's lay theory of something (such as Theory of Mind, “people's theories of intelligence,” or “conspiracy theory”). In the 392 other articles (19.2% of the sample), the string ^*^theor^*^ matched terms that were used to refer to a psychological theory.

Of these 392 articles, 222 articles (56.6%) made only a general reference to theory, either to an unnamed theory (e.g., “a sensorimotor theory of beat induction”), to a class of theories (e.g., “current theories of word learning”), or to theorizing in general (e.g., “recent theory suggests,” “implications for theory and practice,” or “a lack of theoretical grounding”), while 170 articles (43.4%) named at least one theory, such as “attachment theory” or “intentional change theory.” Compared to the proportion of named hypotheses (see Section Hypotheses.) the proportion of named theories was more than twice as high. Most of the articles (138) mentioned only one theory, 25 articles mentioned two theories, and 7 articles mentioned three or four theories. This finding indicates that comparisons of competing theories (analogously to competing hypotheses) were not that common in our sample of articles.

**Figure 2:**
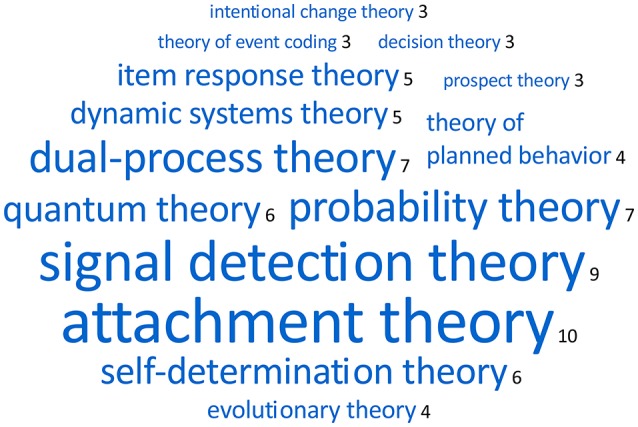
Word cloud of the top 14 theories shared by at least three articles.

Altogether, 139 *different* theories were mentioned; 113 theories in one article only, and 26 theories in two or more articles. The top theories are shown as a word cloud in Figure 2. It should be noted, however, that the frequency with which single theories were mentioned in articles was correlated with the partaking of that article in a thematic *research topic* (i.e., special issue): Of the ten articles referring to *attachment theory*, five were part of the research topic “Parenthood from biology to relation”; of the 13 articles referring to *probability theory* or *quantum theory*, twelve were part of two research topics, “Quantum structures in cognitive and social science” (9) and “Improving Bayesian reasoning: what works and why?” (3), and all three articles referring to *intentional change theory* were part of the research topic “The impact of shared vision on leadership, engagement, and organizational citizenship.”

The large number of singular theories reflects the breadth of the field, but also the extent of fragmentation of the theoretical landscape in psychology. Most theories are restricted to specific phenomena, and appear to be in use in single subfields of the discipline, rather than applied to organize a broad range of phenomena. In fact, of the 392 articles that made a general or specific reference to theory, only 23 referred either to a unifying perspective or to an integration of models, theories, or views from different subfields into broader theoretical frameworks.

#### All six terms

Each of the six terms that were analyzed in the previous sections is in itself an important indicator for theoretical reflections or theory building. A still more inclusive picture of references to theory-related issues is rendered by considering all articles with at least one reference to any of the six terms.

Combining the 269 articles referring to a named or unnamed scientific *hypothesis*, the 12 articles referring to *law* in a scientific meaning, the 214 articles referring to *mechanism* as an explanation for a psychological phenomenon, the 359 plus 24 articles referring to *model* and *simulation* as representing a psychological phenomenon or psychological data, and the 392 articles referring to a named or unnamed *theory*, yields a total of 918 articles, corresponding to 44.9% of the articles in our sample of 2,046 articles.

#### Summary

In the previous sections, we assessed the referents of each match of six strings used in the basic text search, we identified irrelevant meanings, and we scrutinized the usage of the corresponding theory-related terms *hypothesis, law, mechanism, model, simulation*, and *theory*. While the number of matches found in the basic text search represents an upper bound of occurrences of the intended usage of the terms, as argued in section Basic Text Search, the numbers found in the qualitative analyses of selected terms are only marginally lower: References to *theory*, whether general or specific, were found in 19.2% of the 2,046 articles (as compared to 21.1% matches of the string ^*^theor^*^ in the basic text search; cf. Table 3); references to *models* as representing psychological phenomena or data were found in 17.5% of the articles (19.0% for the string ^*^model^*^); references to *scientific hypotheses* were found in 13.1% of the articles (13.8% for the string ^*^hypothe^*^); references to *mechanisms* in an explanatory meaning were found in 10.5% of the articles (10.7% for the string ^*^mechanis^*^); references to *simulation* as any modeling technique used for scientific purposes were found in 2.2% of the articles (2.8% for the string ^*^simulat^*^); and references to *scientific laws* were found in 0.6% of the articles (1.1% for the string ^*^law^*^). The results from the qualitative analyses thus closely follow the pattern from the basic text search. They indicate that theoretical concerns do play a role in our selection of articles (in 44.9% of the articles across the board). However, the theoretical landscape turned out to be fragmented and populated by a large number of different theories.

### Distribution of theory terms over article types and specialties

In the previous section, we looked at six theory-related terms in more detail: scientific *hypothesis, law, mechanism, model, simulation*, and *theory*. We now analyse how the subsets of articles referring to these terms are distributed over the article types and specialties.

#### Distribution of theory terms over article types

Since articles of the type *Hypothesis* & *Theory* are intended to “introduce a testable hypothesis or theory or support already existing theories” (cited from the *Frontiers* homepage), we should expect the proportion of articles mentioning theory-related terms to be particularly high in this category. The same may hold for review articles, as these do not present new data, but either aggregate and interpret data with regard to important questions, hypotheses, or theories in the field or review and evaluate different theoretical accounts. And finally, as empirical research should generally be hypothesis- or theory-driven, we also expected original research papers to include references to theory. For other types of articles, for instance articles that represent an opinion or a comment, we had no specific expectation.

In the following, we analyse how the articles referring to the six scrutinized theory-related terms are distributed over the different *Frontiers* article types. We do this separately for the four subsets of articles referring to the terms *hypothesis, mechanism, model*, and *theory*, respectively (as only these occur in a number that is sufficiently large for such a comparison), and combined for the subset of articles referring to at least one of the six terms (i.e., including *law* and *simulation*). If the references to theory-related terms were independent of the article type, then we would expect the distribution to reflect the base rate of articles of each type (as shown in Table 1). The observed distribution was tested by means of chi-square statistics, first for the distribution of articles over *all* observed article types in the respective subset, and then aggregated over a partitioning into five categories that represent the expectations formulated above: research (consisting of *Original Research* articles only; 1,351 altogether), article types that express a personal view (*Opinion, General Commentary, Perspective*, and *Specialty Grand Challenge*; 294 articles altogether), reviews (*Review, Mini Review*, and *Focused Review*; 180 articles altogether), hypothesis/theory (consisting of *Hypothesis* & *Theory* articles only; 107 altogether), and articles of all other types (114 altogether). In addition to these overall tests, we compared the observed frequency of each type category against its base rate by means of binomial tests.

##### Hypothesis

The 269 articles referring generally or specifically to hypotheses (Section Hypotheses) were distributed over nine article types. Their distribution differed significantly from the base rate, calculated both across the nine article types (χ^2^ = 42.2; *df* = 8; *p* < 0.001) and across the five type categories (χ^2^ = 46.6; *df* = 4; *p* < 0.001). As expected, the categories research and hypothesis/theory were both overrepresented within this subset of articles, whereas the categories personal view and other types were underrepresented (Table 4A). In total, 15.2% of the research articles and 27.1% of the hypothesis/theory articles made a reference to a hypothesis, as compared to 4.8% of the articles with a personal view and 3.5% of other types (mean value: 13.1%; Table 4B).

**Table 4:** Distribution of all articles (base rate) and of five subsets of articles referring to different theory-related terms over five categories of article types.

**Article-type category**	**Base rate^a^ (*N* = 2,046)**	**Hypothesis (*N* = 269)**	**Mechanism (*N* = 214)**	**Model (*N* = 359)**	**Theory (*N* = 392)**	**All 6 terms (*N* = 392)**
**(A) DISTRIBUTION OF THE** ***N*** **ARTICLES OVER THE FIVE GROUPS (in %)**						
RESEARCH	66.03	76.58^+++^	68.22^ns^	67.41^ns^	53.83^↓↓↓^	65.90^ns^
PERSONAL VIEW	14.37	5.20^↓↓↓^	4.67^↓↓↓^	6.69^↓↓↓^	12.24^ns^	8.71^↓↓↓^
REVIEWS	8.80	5.95^ns^	15.89^+++^	11.98^+^	13.78^+++^	11.11^++^
HYPOTHESIS/THEORY	5.23	10.78^+++^	9.35^++^	7.24^ns^	15.31^+++^	9.48^+++^
OTHER TYPES	5.57	1.49^↓↓↓^	1.87^↓↓^	6.69^ns^	4.85^ns^	4.79^ns^
**(B) FREQUENCY (*****n*****; %) OF ARTICLES BASED ON THE TOTAL NUMBER IN THE RESPECTIVE CATEGORY**						
RESEARCH	1,351	206; 15.2%	146; 10.8%	242; 17.9%	211; 15.6%	605; 44.8%
PERSONAL VIEW	294	14; 4.8%	10; 3.4%	24; 8.2%	48; 16.3%	80; 27.2%
REVIEWS	180	16; 8.9%	34; 18.9%	43; 23.9%	54; 30.0%	102; 56.7%
HYPOTHESIS/THEORY	107	29; 27.1%	20; 18.7%	26; 24.3%	60; 56.1%	87; 81.3%
OTHER TYPES	114	4; 3.5%	4; 3.5%	24; 21.1%	19; 16.7%	44; 38.6%
Overall	2,046	269; 13.1%	214; 10.5%	359; 17.5%	392; 19.2%	918; 44.9%

a Total number of articles in the respective category as shown in Table 1. Lower than the expected proportion:

↓↓↓ *≤ 0.001*,

↓↓ *≤ 0.01*,

↓ ≤ 0.05; higher than the expected proportion:

+++ *≤ 0.001*,

++ *≤ 0.01*,

+ ≤ 0.05;

ns *not significant; according to a binomial test with the base rate as test proportion (accurate to six decimal places)*.

##### Mechanism

The 214 articles referring to mechanisms in an explanatory meaning (Section Mechanisms) were distributed over nine article types. Their distribution differed significantly from the base rate, calculated both across the nine article types (χ^2^ = 39.3; *df* = 8; *p* < 0.001) and across the five type categories (χ^2^ = 38.6; *df* = 4; *p* < 0.001). As expected, the categories reviews and hypothesis/theory were both overrepresented within this subset of articles, whereas the categories personal view and other types were underrepresented (Table 4A). In total, 18.9% of the reviews and 18.7% of the hypothesis/theory articles made a reference to “mechanism,” as compared to 3.4% of the articles with a personal view and 3.5% of other types (mean value: 10.5%; Table 4B).

##### Model

The 359 articles referring to models as representing psychological phenomena or data (Section Models and Simulations) were distributed over twelve article types. Their distribution differed significantly from the base rate, calculated both across the twelve article types (χ^2^ = 56.9; *df* = 11; *p* < 0.001) and across the five type categories (χ^2^ = 22.6; *df* = 4; *p* < 0.001). This time, only the category reviews was overrepresented, whereas the category personal view was underrepresented (Table 4A). In total, 23.9% of the reviews made a reference to “model,” as compared to 8.2% of the articles with a personal view (mean value: 17.5%; Table 4B).

##### Theory

The 392 articles referring generally or specifically to theory (Section Theories) were distributed over 13 article types. Their distribution differed significantly from the base rate, calculated both across the 12 article types (χ^2^ = 114.5; *df* = 12; *p* < 0.001) and across the five type categories (χ^2^ = 97.6; *df* = 4; *p* < 0.001). The categories reviews and hypothesis/theory were again overrepresented within this subset of articles, whereas the category research was underrepresented (Table 4A). In total, 30.0% of the reviews and 56.1% of the hypothesis/theory articles made a reference to “theory,” as compared to 15.6% of the research articles (mean value: 19.2%; Table 4B).

##### All six terms

**Figure 3:**
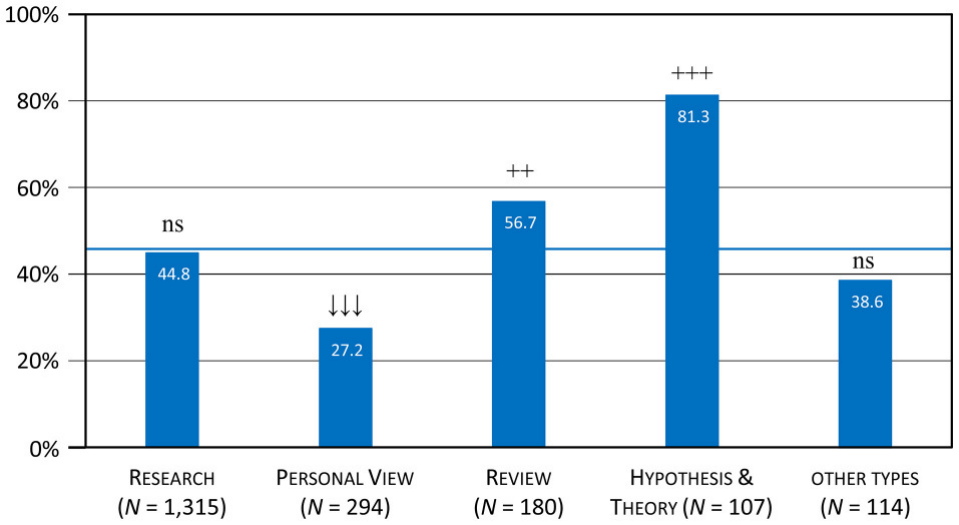
Proportion of article types with theory-related terms (Table 4B, all six terms). The blue line represents the average proportion of 44.9% based on the total of 2,046 articles.

The 918 articles that included a reference to at least one of the six theory-related terms in the title, the keyword list, or the abstract (Section All Six Terms) were distributed over 15 article types. Their distribution differed significantly from the base rate, calculated both across the 15 article types (χ^2^ = 81.3; *df* = 14; *p* < 0.001) and across the five type categories (χ^2^ = 58.7; *df* = 4; *p* < 0.001). The categories reviews and hypothesis/theory were again overrepresented within this subset of articles, whereas the category personal view was underrepresented (Table 4A). In total, 56.7% of the reviews and 81.3% of the hypothesis/theory articles made a reference to at least one of the six theory-related terms, as compared to 27.2% articles with a personal view (mean value: 44.9%; Table 4B and see Figure 3).

In summary, the five categories of article types differed systematically with regard to the number of articles referring to theory-related terms. reviews and hypothesis/theory were above average (as expected) for almost all scrutinized terms, whereas articles with a personal view were below average for most terms. The category research showed a rather mixed pattern: While it was representative overall, the number of articles referring to hypotheses was above average and the number of articles referring to theories below average. Finally, the category other types also showed a representative pattern overall, but the number of articles referring to hypothesis and mechanism was below average. Among the different article formats, the *Frontiers* article type *Hypothesis* & *Theory* and the various review formats therefore seem to most strongly stimulate scientific debate about theories and related issues.

#### Distribution of theory terms over specialties

With regard to the 24 specialties (i.e., subsections of *Frontiers* journals), one might expect that specialties representing basic research have a higher proportion of articles referring to theory and related terms than applied research, which emphasizes the transfer of findings to practice instead (cp. Greenwald, 2012; Stam, 2015). The proportion should be highest, though, for the one specialty with “theory” in its name: *Theoretical and Philosophical Psychology*.

In the following, we analyse how the articles referring to the six scrutinized theory terms are distributed over the different specialties of *Frontiers in Psychology*. We excluded four articles that did not belong to any specialty, resulting in a total of 2,042 articles. Other than this, the analyses were conducted as described in the previous section: first for the four subsets of articles referring to *hypothesis, mechanism, model*, and *theory*, respectively, and then for the subset of articles referring to at least one of the six terms (i.e., including *law* and *simulation*). The observed distributions were tested against the base rate of articles of the various specialties (as shown in Table 2), first over *all* observed specialties in the respective subset, and then aggregated over a partitioning into four categories: specialties that represent basic research (thirteen specialties^4^ with 1,461 articles altogether), specialties that represent applied research (nine specialties^5^ with 470 articles altogether), the specialty concerned with theory/philosophy (i.e., *Theoretical and Philosophical Psychology*, with 23 articles), and the specialty concerned with methods (i.e., *Quantitative Psychology and Measurement*, with 88 articles).

##### Hypothesis

The 269 articles referring generally or specifically to hypotheses (Section Hypotheses) were distributed over 21 specialties. Their distribution did not differ from the base rate, either if calculated across the 21 specialties (χ^2^ = 21.8; *df* = 20; *p* = 0.352) or if calculated across the four categories of specialties (χ^2^ = 5.29; *df* = 3; *p* = 0.152). Across the board, the distribution of articles in this subset followed the base rate of articles; only the category basic research differed from the base rate, and was slightly overrepresented within this subset of articles (Table 5A). In total, 14.2% of the basic research articles made a reference to “hypothesis” (mean value: 13.2%; Table 5B).

**Table 5:** Distribution of all articles (*base rate*) and of five subsets of articles referring to different theory-related terms over four specialty categories.

**Specialty category**	**Base rate^a^ (*N* = 2,042)**	**Hypothesis (*N* = 269)**	**Mechanism (*N* = 214)**	**Model (*N* = 359)**	**Theory (*N* = 392)**	**All 6 terms (*N* = 918)**
**(A) DISTRIBUTION OF THE** ***N*** **ARTICLES OVER THE FOUR CATEGORIES (in %)**						
BASIC RESEARCH	71.55	76.95^+^	81.31^+++^	61.56^↓↓↓^	68.62^ns^	70.15^ns^
APPLIED RESEARCH	23.02	20.07^ns^	14.95^↓↓^	24.51^ns^	24.49^ns^	22.00^ns^
THEORY/PHILOSOPHY	1.13	0.37^ns^	1.87^ns^	0.56^ns^	1.79^ns^	1.42^ns^
METHODS	4.31	2.60^ns^	1.87^↓^	13.37^+++^	5.10^ns^	6.43^++^
**(B) FREQUENCY (*****n*****; %) OF ARTICLES BASED ON THE TOTAL NUMBER IN THE RESPECTIVE CATEGORY**						
BASIC RESEARCH	1,461	207; 14.2%	174; 11.9%	221; 15.1%	269; 18.4%	644; 44.1%
APPLIED RESEARCH	470	54; 11.5%	32; 6.8%	88; 18.7%	96; 20.4%	202; 43.0%
THEORY/PHILOSOPHY	23	1; 4.3%	4; 17.4%	2; 8.7%	7; 30.4%	13; 56.5%
METHODS	88	7; 8.0%	4; 4.5%	48; 54.5%	20; 22.7%	59; 67.0%
Overall	2,042	269; 13.2%	214; 10.5%	359; 17.6%	392; 19.2%	918; 45.0%

a Total number of articles in the respective category as shown in Table 2, excluding the four articles that do not belong to any specialty. Lower than the expected proportion:

↓↓↓ *≤ 0.001*,

↓↓ *≤ 0.01*,

↓ ≤ 0.05; higher than the expected proportion:

+++ *≤ 0.001*,

++ *≤ 0.01*,

+ ≤ 0.05;

ns *not significant; according to a binomial test with the base rate as test proportion (accurate to six decimal places)*.

##### Mechanism

The 214 articles referring to mechanisms in an explanatory meaning (Section Mechanisms) were distributed over 21 specialties. Their distribution differed significantly from the base rate, calculated both across the 21 specialties (χ^2^ = 40.1; *df* = 20; *p* = 0.005) and across the four categories of specialties (χ^2^ = 12.9; *df* = 3; *p* = 0.005). The category basic research was overrepresented within this subset of articles, whereas the categories applied research and methods were underrepresented (Table 5A). In total, 11.9% of the basic research articles made a reference to “mechanism,” as compared to 6.8% of the articles in applied research and 4.5% of those in methods (mean value: 10.5%; Table 5B).

##### Model

The 359 articles referring to models as representing psychological phenomena or data (Section Models and Simulations) were distributed over 22 specialties. Their distribution differed significantly from the base rate, calculated both across the 22 specialties (χ^2^ = 91.7; *df* = 21; *p* < 0.001) and across the four categories of specialties (χ^2^ = 74.8; *df* = 3; *p* < 0.001). This time, the category basic research was underrepresented and methods overrepresented (Table 5A). In total, 15.1% of the basic research articles made a reference to “model,” as compared to 54.5% of the methods articles (mean value: 17.6%; Table 5B).

##### Theory

The 392 articles referring to theory generally or specifically (Section Theories) were distributed over all 24 specialties. Interestingly, their distribution did not differ significantly from the base rate, either if calculated across all specialties (χ^2^ = 29.7; *df* = 23; *p* = 0.158) or if calculated across the four categories (χ^2^ = 2.92; *df* = 3; *p* = 0.404). The distribution of articles in this subset followed the base rate of articles in all categories (mean value: 19.2%; Table 5).

##### All six terms

**Figure 4:**
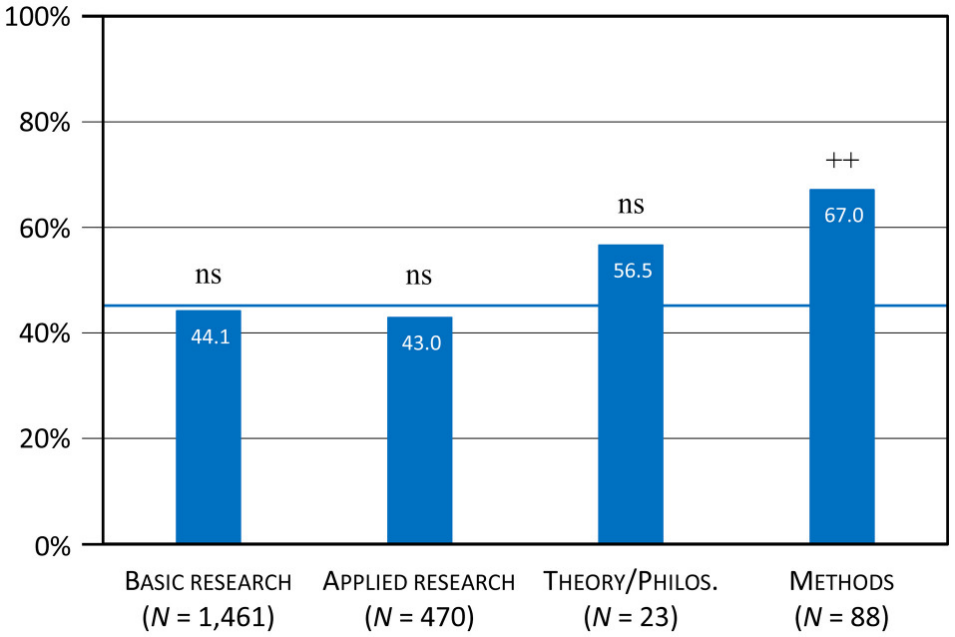
Proportion of specialty categories with theory-related terms (Table 5B, all six terms). The blue line represents the average proportion of 45.0% based on the total of 2,042 articles.

The 918 articles that included a reference to at least one of the six theory-related terms (Section All Six Terms) were also distributed over all 24 specialties. Their distribution did not differ significantly from the base rate if calculated across all specialties (χ^2^ = 34.9; *df* = 23; *p* = 0.053), but if calculated across the four categories of specialties (χ^2^ = 10.9; *df* = 3; *p* = 0.012). Only one category differed from the base rate: methods was overrepresented (Table 5A), with 67.0% of the methods articles making a reference to at least one of the six theory-related terms (mean value: 45.0%; Table 5B and see Figure 4).

In summary, the four categories of specialties showed different patterns regarding the terms to which authors preferentially referred. The category basic research was above average regarding references to hypotheses and mechanisms and below average regarding references to models, showing a representative pattern overall. The category methods revealed a somewhat complementary pattern: below average regarding references to mechanisms, above average regarding references to models, and also above average overall. The categories applied research and theory/philosophy showed almost representative patterns, with only minor deviations from the base rate. Contrary to our expectation, there was little difference between basic and applied research. In fact, the difference between these two categories (tested against the base-rate) was statistically significant only for the term *mechanism* (χ^2^ = 8.67; *df* = 1; *p* = 0.003); in all other cases, it was not significant (χ^2^ < 2.88; *df* = 1; *p* > 0.089). The proportion of references to theory-related terms in articles of the specialty *Theoretical and Philosophical Psychology* is promisingly high (56.5%), but not significant due to the low number of articles in this specialty.

## Discussion

The main goal of this article was to quantitatively assess the role played by theory in contemporary psychology. We addressed this question by using different proxies: We took a sample of journal articles, drawn from the largest online journal, and inspected the titles, keyword lists, and abstracts for a selection of specific theory-related terms. Following a brief summary of our findings, we discuss possible limitations of the proxies and the prospect of theoretical advancement.

### Summary of key findings

We began our analysis with a basic text search for terms related to empiricism and theory in the titles, the keyword lists, and the abstracts of 2,046 *Frontiers* articles. As expected, the frequencies of matches suggest that psychological research—as represented in our sample of papers—is strongly empirical in nature. We also found matches for theory-related search strings, particularly for ^*^theor^*^ and ^*^model^*^, but with lower frequencies compared to terms related to empiricism.

Inspecting the matches for a selection of six theory-related terms revealed that references to scientific *laws* and *simulations* were rare, references to *mechanisms* and *hypotheses* occurred more often, and references to *models* and *theories* most often. Of these, *theories* was the term of prime interest to this investigation, and with an occurrence in 19.2% of all articles in our sample, it is the one theoretical term that is used most frequently: in 10.9% of cases in a general manner (e.g., by making reference to an unnamed theory, a class of theories, or theorizing in general), and in 8.3% of cases more specifically (by making reference to at least one named theory). It should be noted that these are likely rather generous estimates, as we included references to theory in a fairly general sense and also included references to a *lack* of theory. Most of the 170 articles that pointed to specific theories mentioned only one theory (81.2%), and most of the 139 distinct theories that were mentioned were referred to in one article only (81.3%).

Taken together, these findings indicate not only that reference to theory *per se* was not highly frequent, but also that comparison of presumably competing theories was rather rare, reflecting the “widespread but flawed methodological practice of testing only one theory—one's own toothbrush—against data, as opposed to testing two or more theories comparatively” (Gigerenzer, 2010, p. 740). They also indicate that the theoretical landscape is indeed fairly fragmented. One might argue that this lack of theoretical coherence and rigor is partly due to the large range of topics with which psychology is concerned, spanning all the way from psychophysiology, through cognitive, developmental, personality, and social psychology, to applied fields relating to clinical issues, education, or human factors. However, a similar range of topics is covered by biology, where a single theoretical framework such as the theory of evolution still manages both to relate to several subfields from the molecular level in genetics to the systemic relations in the planetary biosphere, and to relate these subfields to each other through its explanatory power.

To obtain a more inclusive assessment of theory-related concerns, we also investigated the occurrence of the combination of six theory-related terms, which increased the proportion of articles comprising any of these entries from about one fifth (max. 19.2%) to more than two fifth (45%). Among the different *Frontiers* article types, two formats particularly foster theoretical considerations: *Hypothesis & Theory* articles and the various *review* formats. For the various subsections (specialties) of the journal, contrary to our expectation, only minor differences were found between basic and applied subsections. The one subsection that deals with theoretical issues in particular—*Theoretical and Philosophical Psychology*—was too small to enable us to draw strong inferences from the data.

### Limitations by proxy

The data we collected for the current analysis made use of four proxies, which may have entailed some drawbacks.

First, we took what psychologists publish in journal articles as a reflection of their concern with theoretical frameworks. The current pressure on rapid proliferation of “least publishable units” (Gigerenzer, 2010; and see Pashler and Wagenmakers, 2012) prompts a trade-off between speed and rigor, which is not genuinely conducive to innovative and in-depth theoretical elaboration. In other words, psychologists might be deeply committed to theoretical advancement, but simply lack the incentives or time to delve into it. Still, as long as such considerations are not published, their existence remains hidden.

Second, we took a sample of more than 2,000 articles, accepted for publication in 2015 in *Frontiers in Psychology*, as a largely representative sample of work in psychological science. This is justified by the fact that this journal is a scientific outlet for articles in *all* fields of psychology, not restricted to specific topics, and in all categories from theoretical papers to applied research. Moreover, due to the lack of space restrictions online, acceptance for publication (subsequent to an interactive review process) is comparatively easier than in classical print journals, giving rise to a substantial amount of publications in a single year. Furthermore, the relevant data for our analysis are openly accessible to everybody. On the other hand, the sample is certainly not entirely representative because not all researchers can afford to use this journal as outlet for their work due to the considerable publication fees for most article types; because it may be more attractive to researchers in some fields of psychology than others (e.g., cognitive psychology was clearly represented in *Frontiers* to a larger extent in 2015 than its relative size would justify); and because researchers may prefer it for the rapid publication of empirical findings, while turning to other, more classical journals or even monographs for theoretical work. Still, the preponderance of publications in specialty sections categorized as pertaining to basic research would likely overestimate rather than downplay the relevance of theory.

Third, we took the title, keywords, and abstract of an article as indicative of the relevance of theory in the article (as compared to the mention of empirical terms). This may not be justified, as authors may have devoted a substantial part of the discussion to theory without explicating this up-front. However, if dealing with theory is so secondary to an article that it is not considered worthy of mention in those parts that advertise its content, disregarding such an article as a major contribution to theory building may indeed appear justified. More often than not, such articles will allude to theoretical frameworks and speculate on theoretical relevance, without necessarily providing new theoretical advances.

In order to gain at least some comparative estimates, we also collected data on the publications in *Cognitive Psychology* in 2015. We chose this journal because it covers the same field as the specialty section of *Frontiers in Psychology* with the largest number of submissions, and because it is considered one of the disciplines' publication flagships due to its theoretical emphasis^6^. Here, the search string ^*^theor^*^ matched in three titles, two keyword lists, and 11 abstracts of a total of 12 of the 33 articles published in 2015 (36.4%), all of which are listed as original research articles. With regard to the usage and meaning of the matching instances, one article referred to an irrelevant meaning (people's lay decision theory), while 11 articles referred either to a named theory or to theory in general (33.3%). This number is significantly higher than the overall rate of 19.2% (cf. Table 5) observed within the *Frontiers* sample (*p* = 0.039; binomial test with the *Frontiers* rate as test proportion), as would be expected from the journal's emphasis on theory. Still, not even half of the articles published there comply with this request. Even if this implied that our search string criterion may have underestimated the factual relevance of theory in the articles in our sample by, say, factor 2, it would still mean that less than half of the *Frontiers* articles explicitly refer to theory.

Fourth, we restricted our qualitative analysis to six terms: *hypothesis, law, mechanism, model, simulation*, and *theory*. While this selection is justified by the relevance of these terms (attested to in classic definitions of theory), by the relation between them (ranging from the description of regularities to the broad theoretical embedding), and by the frequency with which they occurred, the analysis may have missed other relevant concepts. Possible candidates are “framework,” “perspective,” and “approach.” However, the usage of these terms is often broader and less concrete or elaborated than the usage of the term “theory.” One might even argue that part of the theoretical challenge faced by psychology is due precisely to the terminological and conceptual vagueness in using these terms (see, e.g., the “surrogates for theory”; Gigerenzer, 1998, 2010). Insofar as authors may prefer one of these labels over the more canonical one, our analysis *underestimates* the reference to theory.

A related concern applies to theories that are not referred to as “theories,” such as behaviorism, evolution, or psychoanalysis. In order to obtain at least a rough estimate of such misses, we checked (a) all occurrences of the search string ^*^ism^*^ and (b) the 481 unique keywords that occurred in more than three articles. Many occurrences of the search string ^*^ism^*^ referred to “mechanism” (which is covered by our analysis), many others referred to psychological phenomena (e.g., autism, bilingualism, individualism, optimism, and sexism), and some referred to more general scientific positions (e.g., Cartesian dualism, cognitivism, constructivism, empiricism, nativism, postpositivism, and utilitarism). We did not find any reference to “behavio[u]rism.” Among the unique keywords, we found only three instances that referred to theories or classes of theories: “evolutionary psychology” (9 articles), “evolution” (6 articles), and “psychoanalysis” (4 articles). Of these 19 cases, seven were not included in our initial classification as theory-related; including them increases the number of articles referring to named theories from 170 (8.3% of the 2,046 articles) to 177 (8.7%). In other words, even if we may have *underestimated* the frequency of references to a specific theory, this aberration would be rather small. On the other hand, it also *overestimates* it slightly through the fact that in some cases, mentions of the term *theory* actually refer to a *lack* of theory, and not every mention of a (named) theory really involves a critical examination of this theory.

Related to this concern, yet more problematic, is the possibility that researchers may take a specific theoretical framework as their starting point, or as the context in which their work is situated, without explicating it. For instance, most research in social psychology these days is arguably guided by assumptions about cognitive processing, and yet very few authors ever refer explicitly to the information processing paradigm. But while in such cases reference to the underlying theoretical framework may go without saying, this is far from being true in all fields of psychology. For the same detailed phenomenon, competing theoretical accounts may persist for decades, and no set of overarching theories is in sight, even for restricted domains (as indicated by the large number of theories invoked in single articles; see Section Theories). Even the most frequently mentioned theories were cited in less than 0.5% of the articles in our sample. Researchers can therefore not take a specific theoretical approach for granted. Moreover, even in fields where there is more homogeneity overall (e.g., in the domains of information processing, behaviorism, or psychoanalysis), it is unclear which role the respective theory actually plays for the design of a given study or the interpretation of its results. Design and interpretation may simply follow the conventions in the respective field or an established empirical paradigm, and more often than not, the goal will be to modify or refine what we know about boundary conditions rather than to strive for theoretical integration.

And finally, as pointed out by one of the reviewers, theory may also get less attention (in terms of frequency) simply because a great deal of data is required for generating or refining a single theory. Yet, while this asymmetric relation between data and theory is beyond question, we still hold that the relation itself should be made explicit. If a study aims at data that would be informative for a specific theory, its salience and relevance will be strongly increased by reference to this theory.

We therefore believe that, despite the limitations inherent in our proxies, our overall analysis still provides a valid assessment of the (relatively minor) relevance of theoretical concerns as compared to empirical concerns. Evidence in favor of this assessment includes the substantially larger emphasis on empirical than theoretical concepts in the articles sampled here (Table 3), the rather infrequent mention of “theory” and the extensive list of singular theories, most of which are addressed by one article only (Section Theories), and the predominant lack of papers that explicitly address theory unification or integration (for an exception see, e.g., Stam, 2015).

### Prospects of theoretical advancement

As illustrated in the introduction, there is greater consensus on the fact that psychology lacks grand unifying or even merely non-controversial medium-sized theories than there is on whether psychology actually needs such theories in the first place. If one adopts the position that theories come and go, while reliable effects are what really counts (e.g., Simons, 2014), the large number of original research contributions to most psychological journals will be noted with appreciation. The major concern to be tackled, in that case, will be to ensure replicability of data and effects. If, by contrast, one adopts the position that theories are indispensable for making sense of these data (Edelman, 2012; Stroebe and Strack, 2014) and for promoting scientific advancement (Gigerenzer, 2010; Hommel and Colzato, 2017), the picture of the discipline emerging from the previous sections, of relatively few strong theoretical contributions, will be disconcerting.

However, even if one views yet another call for a “unified psychology” with skepticism (for a brief history of such calls and reasons for skepticism, see Stam, 2015), it seems more than clear that *greater* unification along theoretical lines is sorely needed.

On a smaller scale, this would require attempts to resolve the decades-long controversies listed in Greenwald (2012). Some of these are actually getting closer to resolution, be it by conceptual translation, by the recruitment of new and cross-disciplinary methods, or by identifying the boundaries between empirical domains of competing theories. In some cases, an important first step is conceptual clarification and agreement on terminology. To pick an example with which the authors are familiar, the attempt to derive a taxonomy of temporal frames of reference from those used for space has produced eight distinct accounts within a single decade (Bender and Beller, 2014)—a textbook example of Mischel's (2009) “Terminological Tower of Babel.” Despite diverging opinions, a fruitful debate among these scholars would be possible if only they chose to speak the same theoretical language. This would allow them to identify the extent to which they share conceptual ingredients and principles for space-time mapping, and where they actually disagree, thereby preventing a “decades-long wild goose chase” (Edelman, 2012, p. 2). Another step is critical examination of the evidence for each theory, to clear out the “vast graveyard of undead theories,” thereby allowing psychology to overcome accusations of “being little more than opinions with numbers” (Ferguson and Heene, 2012, p. 599).

On a larger scale, this would require attempts to integrate established theories. In those fields of psychology that deal with cognitive topics from an information processing perspective, cognitive architectures like ACT-R (Anderson et al., 2004; Anderson, 2007) provide a powerful theoretical framework, and combining the recognition heuristic model with ACT-R has improved the understanding of how systematic forgetting aids heuristic inference (Schooler and Hertwig, 2005). Another instance of successful theoretical integration is the connecting of fast-and-frugal decision trees with signal-detection theory (Gigerenzer, 2010; Luan et al., 2011). A second natural candidate for such a framework, especially in those fields of psychology with stronger ties to biology, is the theory of evolution (e.g., Jablonka and Lamb, 2007; Edelman, 2012; Levinson and Gray, 2012). A recent upsurge in evolutionary thinking in various related fields—from comparative neurobiology (Lefebvre et al., 2004) all the way to language evolution and cultural change (e.g., Richerson and Christiansen, 2013; Christiansen and Chater, 2016)—attests to its promising possibilities.

As Stam (2015) so aptly recounts, calls for unification in psychology are neither new and nor have they been successful in the long run. Overarching theoretical frameworks, such as behaviorism, cognitivism, or more recently neuroscience, have been embraced with enthusiasm and deserted in disappointment. With this contribution, we do not join in with such calls for *unification*, but rather address theoretical *integration*. Motivated by the conviction that theoretical integration and advancement toward a cumulative science are a legitimate, if not essential goal in every scientific discipline, psychology included, we aimed here to provide empirical data on the state of psychology with respect to this goal. This snapshot, we hope, will help to assess the role that theory currently plays and to raise awareness of the need to improve theory building in practice and in teaching.

## Author contributions

All authors listed have made substantial, direct and intellectual contribution to the work, and approved it for publication.

### Conflict of interest statement

The authors declare that the research was conducted in the absence of any commercial or financial relationships that could be construed as a potential conflict of interest.

## Footnotes

^1^The DOIs ending with 00008, 00217, 00364, 00408, 00413, 00432, 00484, 00518, 00531, 00624, 00699, 00759, 00801, 00833, 00992, 01066, 01561, 01602, 01802, and 01883.

^2^In his survey on the proportion of Nobel Prizes awarded to either developments of theory or method-based contributions, Greenwald (2012) observed that for contributions to theory, the description almost invariably included the word “theory,” whereas contributions to method usually included terms such as “method,” “studies,” or “invention.”

^3^Although, statistical procedures are no obvious candidates for models, we included them here because they share all features with other types of models (i.e., abstraction and a representational relation to a topic of psychological interest). In addition, they come with assumptions, are taken to represent the data (hence posing the question of validity), or used to simulate data and may turn into theoretical metaphors (Gigerenzer, 1991). The extent to which statistical procedures contribute to theory *advancement*, however, is debatable: While some sophisticated statistical procedures explicitly model theories about psychological processes (e.g., Multinomial Processing Tree Models; Erdfelder et al., 2009), the role of more basic, standard procedures is rather to support the decision about a hypothesis, which constributes to theory advancement only indirectly.

^4^The category basic research subsumes the specialties *Cognition, Cognitive Science, Language Sciences, Developmental Psychology, Emotion Science, Personality and Social Psychology, Perception Science, Consciousness Research, Auditory Cognitive Neuroscience, Comparative Psychology, Evolutionary Psychology, and Neuroscience, Decision Neuroscience*, and *Cultural Psychology*.

^5^The category applied research subsumes the specialties *Psychology for Clinical Settings, Educational Psychology, Psychopathology, Movement Science and Sport Psychology, Organizational Psychology, Eating Behavior, Performance Science, Psychoanalysis, and Neuropsychoanalysis*, and *Human-Media Interaction*.

^6^As Greenwald observes, “[p]sychology's most elite empirical journals often oblige authors to establish the value of submitted articles by making clear how their empirical work ‘advances theory”’ (2012, p. 99). This is certainly true for *Cognitive Psychology*, which “specializes in extensive articles that have a major impact on cognitive theory and provide new theoretical advances” (http://www.journals.elsevier.com/cognitive-psychology).
